# Fruit Calcium: Transport and Physiology

**DOI:** 10.3389/fpls.2016.00569

**Published:** 2016-04-29

**Authors:** Bradleigh Hocking, Stephen D. Tyerman, Rachel A. Burton, Matthew Gilliham

**Affiliations:** ^1^Plant Transport and Signaling Laboratory, ARC Centre of Excellence in Plant Energy Biology, School of Agriculture, Food and Wine, Waite Research Institute, University of Adelaide, Glen OsmondSA, Australia; ^2^ARC Centre of Excellence in Plant Cell Walls, School of Agriculture, Food and Wine, Waite Research Institute, University of Adelaide, Glen OsmondSA, Australia

**Keywords:** calcium, fruit ripening, xylem, pectin, water

## Abstract

Calcium has well-documented roles in plant signaling, water relations and cell wall interactions. Significant research into how calcium impacts these individual processes in various tissues has been carried out; however, the influence of calcium on fruit ripening has not been thoroughly explored. Here, we review the current state of knowledge on how calcium may impact the development, physical traits and disease susceptibility of fruit through facilitating developmental and stress response signaling, stabilizing membranes, influencing water relations and modifying cell wall properties through cross-linking of de-esterified pectins. We explore the involvement of calcium in hormone signaling integral to the physiological mechanisms behind common disorders that have been associated with fruit calcium deficiency (e.g., blossom end rot in tomatoes or bitter pit in apples). This review works toward an improved understanding of how the many roles of calcium interact to influence fruit ripening, and proposes future research directions to fill knowledge gaps. Specifically, we focus mostly on grapes and present a model that integrates existing knowledge around these various functions of calcium in fruit, which provides a basis for understanding the physiological impacts of sub-optimal calcium nutrition in grapes. Calcium accumulation and distribution in fruit is shown to be highly dependent on water delivery and cell wall interactions in the apoplasm. Localized calcium deficiencies observed in particular species or varieties can result from differences in xylem morphology, fruit water relations and pectin composition, and can cause leaky membranes, irregular cell wall softening, impaired hormonal signaling and aberrant fruit development. We propose that the role of apoplasmic calcium-pectin crosslinking, particularly in the xylem, is an understudied area that may have a key influence on fruit water relations. Furthermore, we believe that improved knowledge of the calcium-regulated signaling pathways that control ripening would assist in addressing calcium deficiency disorders and improving fruit pathogen resistance.

## Introduction

Fruit are economically important plant organs that face unique challenges in terms of calcium nutrition and physiology. Fruit are architecturally isolated; their supply of water and nutrients changes during fruit development; they often have low rates of transpiration and have low xylem transport rates when compared with the rest of the plant, which limits fruit calcium delivery. We describe how these unique circumstances can create a situation in which calcium deficiencies can easily arise, leading to numerous disorders that impact fruit development and reduce crop quality. Although the strict botanical definition of fruit includes wheat grain and bean pods we mostly restrict ourselves in this review to discussing the multifaceted role of calcium in the flesh-rich seed-associated structures that are commonly referred to as fruit. In particular, this review often uses the role of calcium in grape, tomato, and kiwifruit as a model systems for understanding fruit calcium physiology. Much of our current knowledge on calcium signaling in plants is drawn from specific cell-types such as the guard cell or pollen tube. Different tissues and cell types possess their own protein network, developmental programming and physiology (Henderson and Gilliham, 2015); fruit are not guard cells, mesophyll tissue or pollen tubes – they differ in how they develop and how they respond to stress. Therefore, despite deficiency and toxicity symptoms often being most noticeable in fruit, generally we have a poor understanding of the physiological roles of calcium in fruit development.

The irreplaceable nature of the calcium ion (Ca^2+^) as a signal transduction agent, and in cell wall polysaccharide interactions is undisputed; it is through these processes that calcium is central to stress responses, cell wall growth and remodeling, and to plant tissue development (Dodd et al., 2010; Hepler and Winship, 2010; Kudla et al., 2010; Gilliham et al., 2011b). As Ca^2+^ is such a biologically active ion its concentration and transport must be tightly controlled within plant tissue down to the level of cellular and extracellular compartments. If tissue calcium concentration is high, this can result in cellular toxicity, in overly rigid cell walls and in developmental abnormalities (Conn et al., 2011; Cybulska et al., 2011). When calcium supply is low or transport is disturbed, local calcium deficiencies result. This can lead to membrane breakdown and/or cell wall failure; in fruit this has been proposed to result in disorders such as blossom end rot (de Freitas and Mitcham, 2012). Whether this is the cause of such a disorder or whether calcium deficiency is a result of this condition has been recently debated (de Freitas et al., 2014; Saure, 2014); further insights into how cell wall calcium can influence tissue integrity are provided here.

The cell wall properties of fruit epidermal cell layers are important determinants of pathogen susceptibility. Fruit cell walls are pectin rich, and calcium-pectin cross-links are a major factor in determining the physical and structural properties of fruit. The cell wall is also the source of pectin derived OGAs that elicit pathogen defense responses (Decreux and Messiaen, 2005); cytosolic Ca^2+^ signaling also occurs during defense responses (Dodd et al., 2010), so the interactions between calcium in the cell wall and its cytosolic signaling role warrants further investigation from a fruit-pathogen susceptibility perspective. Treatment of some fruit with calcium-containing sprays is a routine horticultural practice, which can improve cell integrity and disease resistance (Manganaris et al., 2005; Dayod et al., 2010), demonstrating the importance of calcium in determining fruit quality at harvest and improving post-harvest traits. Here, we review the field, and nominate what are the most pressing research questions in this area.

Hormonal controls on cell division and expansion are active in the development of fruit. Many of these phytohormonal pathways utilize changes in cytoplasmic calcium concentration ([Ca^2+^]_cyt_) as a secondary signal messenger (e.g., ABA, jasmonic acid, auxin, GA, ethylene, brassinosteroids, and cytokinins; Fortes et al., 2015). Therefore, the reliance upon Ca^2+^ as a signaling element in a tissue with low and variable calcium supply has been said to create physiological disorders during development, such as blossom end rot in tomatoes (de Freitas et al., 2012). The case for calcium nutrition being an important consideration in establishing normal fruit development and optimizing stress responses is made here. As it is a phloem immobile nutrient, calcium is mainly reliant on transpirational water flow for its accumulation within fruit; however, calcium can regulate water flow through modification of aquaporin activity and cell wall properties that affect cell wall permeability to water so calcium has the potential to affect its own delivery locally (Gilliham et al., 2011b). The influence of calcium in pectin modification and micro-domain gel formation is also a potential source of influencing xylem water transport, water relations and calcium delivery (Zsivanovits et al., 2004). Therefore, the complex relationship between calcium, water, cell walls and signaling pathways make calcium a significant player in fruit physiology and development worthy of further attention.

## Fruit Calcium Transport

Fruit calcium nutrition is dependent upon the physical and molecular pathways of water and calcium delivery, and the impact that calcium signaling can have on cell wall interactions, transpiration, and water transport. The major factors that influence calcium delivery and distribution in aerial tissues include: the rate of xylem water mass flow (as Ca^2+^ is virtually phloem immobile), the competition between ions for binding sites in xylem vessel walls and pit membranes (including H^+^, making pH an important factor), formation of lowly soluble or insoluble complexes (e.g., calcium oxalate) and cellular water/ionic transport mechanisms (Franceschi and Nakata, 2005; Saure, 2005; Gilliham et al., 2011b). Calcium concentration in different cellular compartments can impact water transport processes via membrane-delimited pathways. For instance, increases in [Ca^2+^]_cyt_ can decrease water transport through aquaporins (Alleva et al., 2006; Verdoucq et al., 2008). This has been proposed to affect the relative contribution of apoplasmic and symplasmic water flow and the magnitude of calcium delivery (Gilliham et al., 2011b), with symplastic pathways having a lower capacity for long distance Ca^2+^ movement.

### Long-Distance Calcium Transport

**FIGURE 1:**
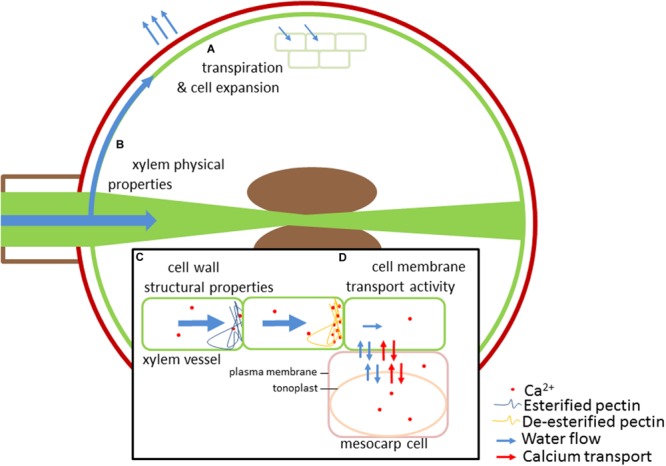
**Several major components determine calcium supply and distribution in fruit.** Calcium is phloem immobile, accumulating through the xylem. Hydraulic factors, particularly fruit water uptake and loss driven by cell expansion and transpiration largely determine the volume of xylem fluid supplied to the fruit **(A)**. Physical properties of the xylem, including xylem vessel development, vessel diameter and connectivity will influence xylem flow into different tissues of the fruit **(B)**. Structural properties of the cell wall, both in xylem vessels and fruit mesocarp and epidermal tissues, can affect the rate of xylem flow and calcium binding in these compartments **(C)**. Pectin is a major component of fruit cell walls; de-esterification of pectin enables formation of calcium crosslinked gels affecting pit membrane porosity, xylem flows and calcium distribution. Additionally, tight regulation of cytosolic calcium concentration occurs through a network of membrane transporters on the plasma membrane and tonoplast, including aquaporins, Ca^2+^-ATPases and CAX ion channels **(D)**. These transport mechanisms can enable accumulation of calcium in particular tissues.

The link between water and calcium transport is particularly apparent when examining sink organs with relatively low transpiration rates, such as those that typically occur in fruit (**Figure 1**). At fruitset the transpiration rate of fruit is at its highest, for instance in kiwifruit this can be as high as 2.3 mmol m^-2^ s^-1^, but this quickly declines to almost a tenth of this value later in development, whereas leaf transpiration is maintained greater than 10 mmol m^-2^ s^-1^ (Montanaro et al., 2014). It is at these early stages of fruit development that most Ca^2+^ is delivered to fruit (Montanaro et al., 2012a,b). In most species the delivery of water, sugar, and basic nutritional inputs during the later stages of fruit ripening occurs largely via the phloem (Drazeta et al., 2004; Rogiers et al., 2006b; Choat et al., 2009). As Ca^2+^ has low phloem mobility, calcium accumulation in aerial sink organs such as fruit is dependent upon its delivery by the xylem (Rogiers et al., 2000; Drazeta et al., 2004). The low phloem mobility of Ca^2+^ can create a situation that leads to localized calcium deficiencies in fruit. The relationship between calcium accumulation, fruit transpiration, and environmental variables is exemplified by observations made in kiwifruit (Montanaro et al., 2014). In kiwifruit, both phloem and xylem appear to contribute to fruit hydration during late development, but their relative contributions are affected by environmental conditions (Clearwater et al., 2012). Under high vapor pressure deficit, kiwifruit calcium accumulation is coupled to transpiration; under low vapor pressure deficit, lower transpiration and fruit water uptake occurs, with calcium accumulation instead more closely coupled with fruit growth rates (Montanaro et al., 2015).

Calcium accumulation in tomato fruit has been shown to be dependent on rates of xylem sap flow, influenced by transpiration and growth rates (Ho et al., 1993; de Freitas et al., 2014). The strength of other calcium sinks in the plant can affect calcium accumulation in the tomato, and may lead to calcium related physiological disorders such as blossom end rot (Ho and White, 2005). ABA treatment of whole plants reduced leaf peduncle xylem sap flow rate and leaf calcium uptake whilst increasing fruit peduncle xylem sap flow rate and fruit calcium uptake; these fruit demonstrated lower susceptibility to development of blossom end rot (de Freitas et al., 2014). However, accumulation of ionic nutrients in fruit is determined not only by water import rates, but also by their relative prevalence and mobility in the phloem and xylem. Unlike, Ca^2+^, which is only xylem mobile, K^+^ is both xylem and phloem-mobile, with K^+^ concentrations in the phloem being up to ten times that found in the xylem (Hocking, 1980). Grape berry potassium accumulation occurs throughout berry development, reaching a maximum uptake rate during early post-veraison with uptake continuing throughout ripening (Rogiers et al., 2006b). In contrast, calcium content (i.e., Ca per berry) generally does not increase after veraison (Rogiers et al., 2006a). The large drop in xylem hydraulic conductance into the berry, which occurs post-veraison, is correlated with a loss of cell vitality and berry shrivel, and also results in a reduction in calcium import (Tyerman et al., 2004; Rogiers et al., 2006b; Tilbrook and Tyerman, 2009). A varietal survey revealed genotype differences in the occurrence of cell vitality loss and berry shrivel in mature grapes (Fuentes et al., 2010). It is not inconceivable that the reduction in Ca^2+^ import could contribute to cell vitality, with those grape varieties that maintain longer periods of water and Ca^2+^ potentially less susceptible to shrivel. The shift away from xylem water delivery during ripening also effectively buffers the fruit against fluctuations in plant water status and water stress events that may affect the plant during ripening (Thomas et al., 2006; Choat et al., 2009). Determining the hydraulic pathways of ionic delivery is vital for understanding patterns of distribution and accumulation and their effects upon fruit development and ripening.

Cation exchange within the xylem plays an important role in Ca^2+^ delivery; CEC is a measure of the abundance of fixed negative charges in the cell wall, a key determinant of the diffusion pattern of cations through the apoplasm. However, studies that have measured the CEC of the xylem are few. The CEC of cell walls for calcium from different root and shoot tissues of *Picea abies* has been measured using transmission electron microscopy energy-dispersive microanalysis. Whilst there was a wide variation between root and shoot tissue CEC was observed, the CEC of the secondary cell wall of xylem tracheids was consistently low (∼24 meq/kg wall material; Fritz, 2007). This suggests that the composition of other zones within the xylem (e.g., pit membranes) and cellular membrane transport mechanisms may also be important for determining Ca^2+^ transport and buffering fluctuations in xylem sap calcium concentration (**Figure 1**).

### Calcium and Hydraulic Conductivity

Compartmentation resulting in high hydraulic resistance in the apoplasm occurs in many tissues. Examples of this include; separation of the extracellular space of the outer root from the root endodermis by the Casparian strip (Nawrath et al., 2013), separation of adjacent xylem conduits by pit membranes (Zwieniecki et al., 2001; Plavcova and Hacke, 2011; van Doorn et al., 2011), separation of the leaf xylem from the leaf apoplasm by bundle sheath cells, and separation of external surfaces of the plant and the underlying apoplasm by the cuticle (Nawrath et al., 2013). Changes in the hydraulic resistance (*R*_h_) of components of the bunch and berry vascular architecture of grapes may account for some of the observed varietal differences in susceptibility to berry shrivel. By studying the *R*_h_ of each component the contributions of particular variables to observed changes in xylem flows may be identified (Tyerman et al., 2004; Choat et al., 2009; Mazzeo et al., 2013). These variables may include; physical barriers (i.e., pit membrane porosity and xylem vessel diameter), structural changes (i.e., formation of pectin gels within the xylem), and cellular water permeability (i.e., through changes in temporal and spatial expression of aquaporins; **Figure 1**). A recent study has demonstrated that hydraulic conductivity of xylem vessels in grape pedicels decreased at veraison and throughout ripening, potentially due to blockages formed by pectin deposition (Knipfer et al., 2015). This effective compartmentation of the apoplasmic space highlights the importance of understanding physical transport barriers as well as cellular transport mechanisms for controlling Ca^2+^ movement and utilization.

A developmental switch to phloem water delivery from predominantly xylem driven delivery reduces the direct hydraulic link of fruit water status to that of the plant (Greenspan et al., 1994). During normal grape development a decrease in mesocarp turgor coincides with the onset of veraison, indicative of phloem solute unloading. Water stress can also cause a drop in fruit mesocarp turgor; however, after veraison, berry mesocarp turgor does not appear to respond to vine water deficit (Thomas et al., 2006). When pre-veraison berries were physically boxed to restrict veraison associated cell expansion, both sugar accumulation and the drop in mesocarp turgor pressure were delayed. When the box was ventilated to allow transpiration, delayed sugar accumulation was not observed and the mesocarp turgor drop was less delayed (Matthews et al., 2009). This suggests that fruit transpiration is required to assist in phloem sugar loading into fruit (by removing excess water; Lang and Thorpe, 1989), and that ripening related changes in mesocarp cell turgor pressures are linked to both rapid cell expansion and sugar accumulation (Matthews et al., 2009). Calcium is involved in the regulation of cell expansion and elongation during pollen tube tip growth through dynamic pectin binding (Jiang et al., 2005; Rounds et al., 2011), binding signaling proteins and modifying ion channel activity (Konrad et al., 2011). Calcium may also be involved in the changes in cell turgor pressure and cell expansion observed during the progression of fruit ripening. The relative contributions of turgor and cell wall changes to fruit softening are still a major point of discussion. However, it is clear that both factors contribute to the onset and development of ripening processes in fruit through complex interactive pathways and feedback mechanisms.

Fruit water relations and ripening-linked shifts in fruit hydraulic conductance vary between species. Kiwifruit (*Actinidia chinensis)* maintains positive water fluxes from both the phloem and xylem into the fruit throughout development, with each pathway contributing approximately equally to the water balance (Clearwater et al., 2012). However, when grown in high vapor pressure deficit conditions *A. chinensis* var. chinensis ‘Hort16A’ exhibits late ripening shrivel, similar to the phenomenon observed in Shiraz grapes. The high surface conductance and transpiration rate observed in Hort16A may cause an imbalance between water delivery to the fruit and transpiration losses (Clearwater et al., 2012). Additionally, kiwifruit does not accumulate sugars until late in the ripening phase; this difference may explain its ability to maintain xylem flow from the plant into the fruit throughout development. A study of kiwifruit xylem hydraulic resistance (*R*_h_) throughout development using pressure chamber and flow meter techniques showed a general increase in *R*_h_ during the second half of fruit development, consistent with previous reports in grapevine and kiwifruit (Tyerman et al., 2004; Choat et al., 2009; Mazzeo et al., 2013). However, the increase in *R*_h_ began prior to ripening, indicating that decreasing xylem inflows in kiwifruit may be attributable to increasing xylem hydraulic resistance (Mazzeo et al., 2013). This contrasts to observations in grape; xylem flow rates into the berry drop around veraison whereas increases in *R*_h_ are observed after veraison (Tyerman et al., 2004; Choat et al., 2009). The parallel use of pressure chamber and flow meter techniques (Mazzeo et al., 2013), and an evaporative flux method (Clearwater et al., 2012), showed differences in the magnitude of resistance measured depending on the methodology employed. The flow meter technique may also underestimate xylem resistance, with the calculations used for estimation of berry hydraulic isolation and the potential for xylem backflow being questioned (Mazzeo et al., 2013). Despite difficulties in accurately and consistently measuring hydraulic resistance, it is highly likely that differences in xylem sap ionic composition and xylem physical properties will contribute to fruit water relations.

### Interactions between Membrane Transport and Fruit Calcium Physiology

The influence of transport proteins on the long distance transport of Ca^2+^ has been reviewed previously (Gilliham et al., 2011b), and varies at both the inter- and intra-species level (White, 2001; Cholewa and Peterson, 2004; Conn et al., 2012); in grapevine the choice of rootstock has also been shown to influence shoot accumulation of calcium (Kidman et al., 2014). The presence of a suberized endodermis limits root apoplasmic flow making symplastic transport a necessity, and the dominant pathway of root xylem loading at low transpiration. Regulation of Ca^2+^ transport across the plasma membrane and organellar membranes is tightly controlled by the expression pattern, interaction and post-transcriptional control of many Ca^2+^ transporters (Kudla et al., 2010; **Figure 1**). For example, the differentially regulated expression of a number of membrane ion transporters is responsible for cell-specific calcium accumulation patterns in plants (Conn and Gilliham, 2010; Conn et al., 2011; Gilliham et al., 2011a). The use of both cell-specific ion and transcript profiling and of genomic and transcriptional natural variation amongst varieties of certain plant species has been useful in the identification of these transporters (Conn et al., 2012). In *Arabidopsis*, knockout of the vacuolar Ca^2+^/H^+^ antiporters AtCAX1 and AtCAX3 resulted in lower mesophyll Ca^2+^ sequestration and higher apoplasmic Ca^2+^, with physiological impacts ranging from reduced stomatal aperture, stomatal conductance and CO_2_ assimilation to reduced cell wall extensibility and leaf growth rate (Conn et al., 2011). Constitutive expression of sCAX1, the *Arabidopsis* vacuolar calcium transporter with its auto-inhibitory region removed, in transgenic tomatoes, increased fruit calcium concentration and vacuolar Ca^2+^ transport (Park et al., 2005). Interestingly, susceptibility to blossom end rot was also increased in these transgenic lines (Park et al., 2005; de Freitas et al., 2011). The constitutive expression of the sCAX1 increased vacuolar calcium accumulation, depleting pools of apoplasmic and cytosolic Ca^2+^, causing increased membrane leakage and blossom end rot (de Freitas et al., 2011). Although some calcium transport mechanisms have been investigated in fruit, calcium signaling in fruit has not, so the broader impact of calcium nutrition, transport and signaling pathways on fruit development and ripening is still largely unknown.

Plants tightly control cellular Ca^2+^ transport in order to keep [Ca^2+^]_cyt_ within the range (∼0.1–10 μM) required for signal transduction (Evans et al., 1991; White, 2000; Dodd et al., 2010). Regulated fluctuations in [Ca^2+^]_cyt_ form the “calcium signature” which is a major determining factor in the specificity of downstream transcriptional and physiological responses (McAinsh and Pittman, 2009; Dodd et al., 2010; Kudla et al., 2010). Different environmental stimuli create specific calcium signatures in particular cell-types (Kiegle et al., 2000; Dodd et al., 2010; Marti et al., 2013). The channels responsible for regulating these calcium transients are still largely unknown, with progress having been reviewed by Swarbreck et al. (2013). Electrically induced calcium transients with different amplitudes and frequencies were shown to induce distinct patterns of gene expression (Whalley and Knight, 2013), indicating that environmental stimuli can translate into specific expression profile changes in calcium signaling components. There is considerable evidence indicating that Ca^2+^ signaling transients also occur in compartments other than the cytosol, e.g., the nucleus, chloroplasts and the apoplasm (Johnson et al., 1995, 2006; Tang et al., 2007; McAinsh and Pittman, 2009). However, these mechanisms are not nearly as well characterized as the cytosolic pathways. Furthermore, Ca^2+^ transient signaling in fruit specific cell types has not been studied. Generic models for how transients are developed in plant tissue and which transporters are involved in their generation are illustrated in reviews such as Kudla et al. (2010) and de Freitas and Mitcham (2012), these are also useful in the context of understanding the nutritional fluxes of Ca^2+^ and how these may affect compartmentation of Ca^2+^ apoplasmically, in the cytoplasm and intracellularly.

Increases in apoplasmic calcium can result in increases in [Ca^2+^]_cyt;_ this has been used to control the duration and amplitude of [Ca^2+^]_cyt_ oscillations in stomatal guard cells to affect guard cell closure (Allen et al., 2001; Webb et al., 2001). *In planta* manipulation of apoplastic calcium ([Ca^2+^]_apo_) can reduce CO_2_ assimilation and transpiration rate, through reducing stomatal aperture (Conn et al., 2011). Some components of an extracellular calcium-sensing pathway have been described; where a plastid localized calcium sensor protein (CAS) mediates stomatal closure in response to changes in extracellular calcium (Han et al., 2003; Wang et al., 2012). Antisense *cas* lines showed reduced water use efficiency and photosynthetic electron transport rate, due to reduced control of stomatal aperture and transcription of electron transport components (Wang et al., 2014), demonstrating the importance of the extracellular calcium signaling pathways in optimizing photosynthesis and water use. The supply of Ca^2+^ to fruit is dependent upon transpirational water flow and storage rate (i.e., Ca^2+^ transport into the vacuole via CAX transporters; Conn et al., 2011), therefore [Ca^2+^]_apo_ in both leaves and fruit are likely to have an impact on Ca^2+^ supply; furthermore, high [Ca^2+^]_apo_ in fruit will directly regulate [Ca^2+^]_cyt_, cell wall properties, gene expression and water relations of the fruit, but the impact that this has on fruit quality outcomes at harvest and during storage is totally unexplored.

Characterization of changes in apoplasmic and vacuolar solute composition that supply grapes supports the notion of a switch from symplasmic to apoplasmic unloading of phloem solutes during late ripening. The table grape variety Concord maintains high apoplasmic pH (relative to vacuolar pH) late into ripening, whereas, in the shrivel susceptible variety Merlot the pH difference between these compartments is reduced to zero during late ripening, indicating a loss of membrane selectivity in this variety (Keller and Shrestha, 2014). This is supported by recent measurements of electrical impedance in Shiraz berries (Caravia et al., 2015). The switch to apoplasmic phloem unloading enables accumulation of high sugar levels in ripening fruit but also modifies the conditions of the apoplast with potential impacts on cell wall modification and calcium binding. Merlot demonstrates a dramatic jump in apoplasmic glucose and fructose concentrations during the transition from red to ripe berries (Keller and Shrestha, 2014). The accumulation of sugars in the apoplasm activates cell wall localized invertases and hexose/proton transport pathways in berries (Hayes et al., 2007). The loss of cell turgor, vitality, and membrane integrity in the locular tissues during ripening may be related to apoplasmic unloading and the ongoing accumulation of solutes from the adjacent central vasculature (Tyerman et al., 2004; Krasnow et al., 2008). However, the onset of berry death normally occurs after the transition to apoplasmic unloading. Additionally, cell membrane capacitance in the berry is maintained through the cell death phase, indicating intact membranes (Caravia et al., 2015). This suggests that rather than ‘cell death,’ the loss of cell vitality often observed may actually represent a loss of membrane selectivity allowing distribution of some solutes (e.g., sugars, ions, and perhaps the cell vitality stain fluorescein diacetate) into the apoplasm. The effect of solute accumulation in the apoplasm and associated changes in cell turgor on fruit water relations requires further investigation.

## Calcium-Cell Wall Interactions During Fruit Development

The cell wall is composed of a diverse array of complex polysaccharides. In dicots, the primary cell wall consists of cellulose microfibrils bound in a matrix of pectins and hemicelluloses. The Cellulose is extruded through the plasma membrane by cellulose synthase complexes, whereas pectins and hemicelluloses are synthesized within the Golgi apparatus, and are transported to the cell surface where further synthesis and modification may take place (Gendre et al., 2013). The matrix polysaccharides are very diverse in their composition, with a variety of sugar residues, linkages and side chains present; their synthesis and modification is therefore accomplished by a large number of genes (Burton et al., 2010). The cell wall is a dynamic structure that responds to both developmental and environmental stimuli by structural remodeling; environmental perturbations include pathogen attack, light, and touch (Hoson, 1998; Seifert and Blaukopf, 2010). Cell wall modifying enzymes activated at different stages of development, and under certain conditions (e.g., heat, pH changes in the apoplasm), are responsible for modification and degradation of cell wall polysaccharides (Grignon and Sentenac, 1991; Brummell, 2006). The chemical changes that occur in fruit cell walls during development include; modification of pectin side chains, depolymerisation of pectins, and degradation of xyloglucan (a hemicellulose), and the activity of non-catalytic proteins such as expansions and AGPs. Together with other ripening related processes (such as the accumulation of solutes) this leads to a number of physical and textural changes in fruit that can help us to classify different types of fruit by their ripening mechanisms. Physical changes in fruit cell walls are associated with ongoing modification and solubilisation of pectins; calcium-pectin cross-links are a key factor in determining pectin physical properties.

### General Calcium and Pectin Interactions

Pectins are a complex family of polysaccharides that are structurally related by the occurrence of (1,4)-α-linked galacturonan in the backbone, commonly as homogalacturonan, or as the rhamnose/galacturonan disaccharide repeat rhamnogalacturonan-I (Mohnen, 2008). The galacturonan residues of the backbone may be methyl-esterified or acetylated; homogalacturonan is secreted into the cell wall in an esterified form (Willats et al., 2001). A wide variety of linear and branched side chains are also observed, forming the pectin structural classes rhamnogalacturonan-II, xylogalacturonan, and apiogalacturonan. Rhamnogalacturonan-II is the most complex pectin, it can include up to 12 different sugar residues and more than 20 different linkage types. These have been reviewed previously (Vorwerk et al., 2004; Mohnen, 2008; Burton et al., 2010). The structural complexity of pectin, driven by the expression of a range of pectin synthesizing and modifying enzymes throughout development, implicates pectin in an array of potential interactions and functional roles.

The prevalence of ionic and ester bonds between adjacent pectins play an important role in the physical properties of fruit cell walls. These bond interactions influence the solubility of pectins. Suitable ions for cross-linking adjacent pectins include calcium-forming junctions between de-esterified homogalacturonans and boron forming di-ester bonds between rhamnogalacturonan II units. Associations between adjacent homogalacturonans ionically linked by calcium ions have been characterized as forming an “egg-box” structure. Although this structure has been demonstrated in pectin extracts (Tibbits et al., 1998), the diversity of side chains and modifications within the pectic polysaccharides, as well as the complexity of other cell wall components makes such interactions difficult to characterize *in planta*. The importance of pectin structure for determining the hydraulic and elastic properties of pectin gels has been examined mostly *in vitro*; understanding the complexity of these cell wall interactions *in planta* requires further research.

**FIGURE 2:**
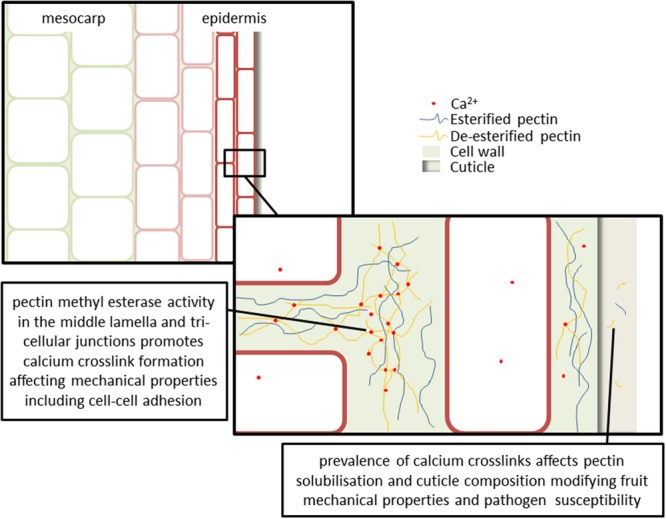
**Cell wall changes and calcium-pectin crosslink formation affects fruit mechanical properties, water relations and pathogen susceptibility.** Upregulation of cell wall modifying and hydraulic regulatory genes (e.g., pectin methyl esterases, polygalacturonases, and aquaporins) occurs in the mesocarp at veraison. The majority of pectins occur in the middle lamella, with smaller amounts observed in the primary cell wall. Localization of calcium in particular extracellular domains is hypothesized to affect cell wall loosening and cell separation. This may be particularly relevant at three way cell junctions where turgor pressure is driving the separation of cells and the formation of large intra-cellular spaces. Cuticle composition is an important variable in determining both fruit physical properties and transpiration water losses throughout berry development. Processes affecting polysaccharide solubilisation and movement into the cuticle, such as pectin de-esterification and calcium crosslinking, and production of oligogalacturonides will modify fruit mechanical properties and pathogen susceptibility.

The majority of pectins occur in the middle lamella (outermost part of the extracellular matrix; where cell junctions occur), with smaller amounts observed in the primary cell wall (Lee et al., 2011). Micro-domain localization of calcium in particular extracellular domains is hypothesized to affect cell wall loosening and cell separation (**Figure 2**). This may be particularly relevant at three way cell junctions where turgor pressure is driving the separation of cells and the formation of large intra-cellular spaces (Willats et al., 2001). It has been suggested that the major physical effects of pectin modification will therefore be in cell-cell adhesion rather than strength of the primary cell wall (Ferguson, 1984). However, species and tissue differences in patterns of pectin deposition and modification (through controlled expression of an array of cell wall modifying enzymes) indicate that the situation may be much more complex.

Cell wall acidification promotes cell growth and expansion by displacing pectin-bound calcium through protonation of pectin carboxyl groups. The pH of the apoplasm may be affected by the pH of the xylem sap when water delivery is high, depending on the buffering capacity of the xylem solutes. Control of apoplasm pH occurs through the activity of the plasma-membrane localized H^+^-ATPase and is buffered by the CEC of the apoplasm. When exposed to high concentrations of NaCl in the growth media a decrease in leaf growth rate of a salt-sensitive maize cultivar is correlated with a reduction in H^+^-ATPase activity, resulting in increased apoplasmic pH whilst a tolerant hybrid cultivar showed none of these effects (Pitann et al., 2009). This finding is contrasted by work measuring ion fluxes in bean leaf, which showed H^+^ eﬄux from the mesophyll upon addition of NaCl directly to the mesophyll (Shabala, 2000), although this may be in part related to the displacement of H^+^ from the cell wall by Na^+^. Amelioration of the effects of salinity on growth by high calcium has been observed; this may result from interactive effects with plasma membrane transport proteins (such as H^+^/cation exchangers), or through a reduction in the rate of Ca^2+^ displacement from pectin cross links by Na^+^ and H^+^ ions (Shabala, 2000). Additionally, cell wall localized expansions show optimal activity at low pH. They are believed to act by reducing hydrogen bonding between primary cell wall components, allowing slippage between adjacent polysaccharides and hence cell wall expansion (Sampedro and Cosgrove, 2005; Dal Santo et al., 2013). Thus, changes in apoplasmic pH can have significant effects on the dynamics and composition of the cell wall.

### Calcium, Pectin, and Fruit Softening

Fruit softening is often attributed to changes in the composition of the cell wall, and particularly to the impact of pectin de-esterification and calcium crosslink formation on cell wall physical properties including strength and elasticity, cell wall loosening and swelling (**Figure 2**). Changes in [Ca^2+^]_apo_ and the secretion and modification of pectins are important for the physical development of fruit. Some species (e.g., strawberry and plum) exhibit cell wall swelling during ripening which results in a soft textured fruit, whereas other species (e.g., watermelon and apple) do not exhibit swelling and maintain crisp textured fruit (Redgwell et al., 1997).

Throughout fruit ripening, pectin de-esterification occurs by the action of PMEs. This exposes the carboxyl residues that can be cross-linked by calcium. The level of PME activity and Ca^2+^ availability within the apoplasm has a direct impact on cell wall strength and expansion (Conn et al., 2011). Studies in grapes suggest that PME expression begins before veraison and continues throughout ripening (Barnavon et al., 2001; Schlosser et al., 2008; **Figure 2**). Mesocarp and skin tissues exhibit different patterns of PME expression in grapes (Nunan et al., 1998; Schlosser et al., 2008; Lacampagne et al., 2010). During the initiation of ripening a raft of cell wall modifying and hydraulic regulatory genes in grape (including expansions *EXP3* and *EXPL*, pectate lyase, a pectin methyl esterase, and aquaporin PIP2;1) are upregulated (Schlosser et al., 2008). This occurs initially in mesocarp; the delayed activation of these genes in the skin suggests a role for the skin in moderating berry growth during ripening. The degree of pectin de-esterification also varies between varieties (Ortega-Regules et al., 2008).

The expression and activity patterns of cell wall modifying enzymes in grapes change throughout development, as well as varying between varieties. In both Cabernet Sauvignon and Semillon, polygalacturonase activity appears correlated with ABA levels, reaching a maximum at veraison (Deytieux et al., 2005). Polygalacturonase activity in skin was not detected, however, transcripts of two isoforms showed different expression patterns, with a common feature being greater expression late in development (100 days after anthesis), indicating a variety of roles for the polygalacturonase family, and a concerted role in cell wall disassembly at maturity (Deytieux-Belleau et al., 2008). However, other research has also indicated that transcript expression of polygalacturonases does not necessarily translate to detectable enzyme activity (Nunan et al., 2001). *In vitro* calcium has an inhibitory effect on polygalacturonase activity; a reduction in calcium concentration and availability following veraison may be linked to a concurrent increase in polygalacturonase activity (Cabanne and Doneche, 2001). In grape skin tissue pectin methylesterase is present throughout ripening with enzyme activity reaching a peak at the beginning of veraison, then decreasing sharply in the subsequent 10 days and increasing steadily thereafter (Deytieux-Belleau et al., 2008). Low levels of polygalacturonase and pectate lyase activity are observed during some stages of ripening; however, hormonal cues may regulate the targeted expression of specific isoforms to drive the depolymerisation of pectin observed during ripening (Nunan et al., 2001).

In addition to the de-esterification of pectins observed in fruit cell walls during ripening, depolymerisation of pectins to shorter sub-units is also an important factor. By using a chelator (e.g., CDTA) followed by size exclusion chromatography to extract and characterize ionically bound pectins, the diversity in timing and degree of depolymerisation that occurs between species can be observed. Some species show almost no change in pectin composition or solubilisation throughout development (e.g., capsicum), whilst others (e.g., tomato) show high degrees of depolymerisation and high levels of chelator soluble pectins (i.e., high levels of calcium bound pectins; Brummell, 2006). This depolymerisation is achieved through the action of polygalacturonases and pectate lyases. Polygalacturonases are absent or detected at very low levels in the fruit of some species which may account for some of the differences in levels of pectin solubilisation. Additionally, it has been demonstrated that reduced expansion activity (normally responsible for loosening of xyloglucan and cellulose networks in the primary cell well during cell expansion) decreases solubilisation of primary cell wall pectins, possibly through reduced access of polygalacturonases to their substrates in this space (Brummell et al., 1999). This finding suggests that it is important to consider more than just transcript levels or enzyme activity when assessing potential for degradation of particular components; calcium availability and activity of other cell wall enzymes may influence substrate accessibility.

The combinatorial effects of pectin modifying enzyme activity, apoplasm pH and calcium concentration determine various mechanical properties of pectin gels including compressive strength, water holding capacity, porosity, and elasticity (Tibbits et al., 1998; Willats et al., 2001; Ngouemazong et al., 2012). The strength of calcium crosslinks is pH dependent, with the strongest bonds forming at apoplasmic pH 6–7. Formation and dissolution of pectin gels by calcium crosslinks is highly dependent on the level of de-esterification (i.e., available carboxyl groups) and free calcium ion concentration (Tibbits et al., 1998). Gel swelling can be observed during cell wall dissolution, due to both the osmotic pressure created by free carboxyl groups in the pectin matrix (occurring when ionic strength is low), and the disassociation of calcium cross-linked pectins. This can be expressed as the ratio of free calcium ions to carboxyl groups (i.e., if [Ca^2+^] _free_: COO^-^ < 0.05 significant swelling is likely to occur). As gel dissolution and swelling occurs, the breakdown of calcium crosslinks reduces the stiffness of the gel. Swelling of a gel is generally at maximum around pH 3, which is also the pH at which calcium crosslinking and gel shear strength are at a minimum (Tibbits et al., 1998). Reported work with pectin concentrations similar to those observed in plant cell walls (films with ∼30% pectin), demonstrate that pectin hydration status (or degree of swelling) has a linear inverse relationship with tensile strength (Zsivanovits et al., 2004). Additionally, the hydraulic properties and susceptibility to swelling of the pectin matrix are determined by both the pectin composition and the ionic composition of the space (Zsivanovits et al., 2004). Through understanding the pectin ion interaction effects on gel properties *in vitro* it is likely we will advance our comprehension of fruit ripening processes.

### Calcium, Pectin and Pathogens

Plant and fungal PMEs have different modes of function; plant PMEs generally operate in a blockwise manner, de-esterifying multiple homogalacturonan residues along a single chain, whereas fungal PMEs operate in a non-blockwise manner (Willats et al., 2001). Patterning of pectin modification and calcium binding may affect the attachment and rate of pectin cleavage by polygalacturonases (**Figure 2**). Three botrytis (*Botrytis cinerea*) isolates exhibited calcium inhibition of polygalacturonase activity. The calcium concentration required to inhibit enzyme activity varied between isolates (Chardonnet et al., 2000). The pathogenicity of these isolates also varied between four apple varieties, indicating that the interaction between the pathogen cell-wall degrading enzymes and the composition of the fruit cell wall is important for determining pathogenicity (Chardonnet et al., 2000). It has been demonstrated that the calcium content of grape skin cell walls is negatively correlated with susceptibility to botrytis enzymatic digestion (Chardonnet and Doneche, 1995). Calcium infiltration reduced the level of pectin degradation by botrytis in grapes (Chardonnet et al., 1997), and reduced the level of decay in apples (Chardonnet et al., 2000). Complex interactions between calcium nutrition and the diversity of pectin profiles seen in different species, varieties, tissues, organs, and developmental points influence susceptibility to fungal pathogens. These studies indicate that calcium treatments may be worthwhile exploring as a management option for some fruit pathogens (Dayod et al., 2010).

Degradation of pectic homogalacturonan backbones generates short chain molecules known as OGAs; these have been implicated in pathogen defense signaling activation. This role is carried out through OGA binding by the wall-associated kinase (WAK) family (Decreux and Messiaen, 2005). It is likely that functional OGAs may be prevalent in fruit, and often affect ripening, as ripening fruit has high pectin content and is attractive to a variety of pathogens. Many factors influence the defense response eliciting capacity and specificity of OGAs, including; calcium availability, length of OGA, degree of methyl-esterification and degree of acetylation (Decreux and Messiaen, 2005; Vallarino and Osorio, 2012; **Figure 2**). The extracellular domain of *Arabidopsis* WAK1 binds OGAs only in the presence of calcium and calcium crosslink forming conditions (Decreux and Messiaen, 2005). Transgenic expression of a fruit-specific PME from cultivated strawberry in wild strawberry (*Fragaria vesca*) resulted in a modified pattern of OGA esterification in the transgenic fruit. This change was sufficient to constitutively activate defense responses in the transgenic plant, thereby increasing botrytis resistance (Osorio et al., 2008). A variety of evidence suggests that, in addition to WAKs binding specific OGAs during pathogen responses, they also bind cell wall pectins during normal development to regulate cell expansion (e.g., reduction in *WAK* expression via antisense has been shown to reduce cell size) reviewed in Kohorn and Kohorn (2012). It is apparent that the specificity of calcium-pectin-WAK interactions may facilitate multiple signaling pathways important in pathogen defense activation as well as during the normal developmental control of cell expansion.

### The Influence of the Cuticle

Changes in cuticle composition (e.g., relative abundance of polysaccharides) can affect fruit mechanical properties and transpiration rate. Fruit cuticles are typically thicker (but also more water permeable) than leaf cuticles; the scarcity of stomata on fruit also suggests that cuticle composition is important for fruit water relations (Martin and Rose, 2014). A study modeling the impacts of environmental variables on kiwifruit transpiration revealed both seasonal and diurnal variation in transpiration rates, with skin conductance being the key fruit variable in determining fruit transpiration rates (Montanaro et al., 2012b). A tomato cultivar (‘Delayed Fruit Deterioration’) with altered cuticle architecture was shown to have low fruit transpiration and increased cell turgor pressure, leading to delayed softening despite undergoing normal ripening related cell wall modifications (Saladie et al., 2007), and application of gibberellins was shown to increase cuticle thickness in tomato (Knoche and Peschel, 2007). In grapes (cv. Riesling), a drop in the transpiration permeability of the cuticle occurs from pre-veraison to post-veraison (Becker and Knoche, 2011), and this drop is strongly correlated with increased cuticle deposition (Becker and Knoche, 2012). Indeed, recent work has identified both varietal differences and developmental changes in the cuticular conductance of grape berries, possibly attributable to cuticle composition (Keller et al., 2015). The composition of the cuticle changes throughout development in cherry tomato (cv. Cascada); cuticle mass per unit fruit surface area increased rapidly from 10 days after anthesis to reach a maximum 15 days after anthesis (subsequent increases in cuticle thickness were attributed to reduced cuticle density; Dominguez et al., 2008). Interestingly, another study in the same cultivar looking at the cuticle mechanical properties found a shift from elastic to predominantly viscoelastic behavior from 10 to 15 days after anthesis. These changes in the cuticle mechanical properties were correlated with the ratio of cutin:polysaccharide present; high ratios were associated with cell enlargement growth stages, and lower ratios (approaching 1:1) were associated with stages where cell expansion is minimal (i.e., early cell division and later ripening phases; Espana et al., 2014). As such, it is hypothesized that polysaccharides in the cuticle contribute elastic properties, and cutin confers viscoelastic properties. It is clear that cuticle composition is an important variable in determining both fruit physical properties and transpiration water losses.

## Calcium–Hormone Interactions During Fruit Development

Calcium is a secondary messenger during hormone signaling. Calcium is known to participate in GA, auxin, and ABA signaling to regulate fruitset, initiation of ripening, cell division, cell expansion, and fruit softening (Ferguson, 1984; Saure, 2005; Yu et al., 2006). Additionally, hormonal regulation of cell expansion, cell wall modification, xylem development, and sugar unloading from the phloem can affect calcium distribution within the fruit (Saure, 2005; de Freitas et al., 2014). Although the physiological pathways and interactions of plant hormones and calcium are still being uncovered, many hormone and calcium treatments are already used for horticultural improvement. The role of plant hormones in fruit development and ripening processes has been extensively reviewed (Ruan et al., 2012; McAtee et al., 2013; Osorio et al., 2013; Wang and Ruan, 2013; Kumar et al., 2014; Leng et al., 2014). As both are components of an array of complex signaling pathways, the accumulation and activity of calcium and phytohormones is tightly controlled at the tissue level. Subsequently, perturbed calcium nutrition may create multiple plant hormonal responses that are difficult to characterize. This section will therefore articulate the current knowledge and gaps in our understanding of calcium and hormone interactions in fruit.

### Auxin

Auxin has key roles in fruitset, cell division and cell expansion. These developmental pathways both utilize calcium as a secondary messenger and affect patterns of calcium distribution. Fruitset and early development are triggered by auxin synthesis in the ovules during fertilization, which induces GA synthesis, reviewed in Kumar et al. (2014). GA signaling in the pericarp of *Arabidopsis* fruit has been demonstrated to activate a pathway degrading the growth inhibiting DELLA proteins (Fuentes et al., 2012; Kumar et al., 2014). The relationship between GA and auxin is complex, with a GA independent pathway for fruitset being demonstrated in tomato (Serrani et al., 2008; McAtee et al., 2013). High levels of GA are commonly associated with rapid cell expansion and this has also been linked to low or reduced calcium concentrations by disrupting calcium transport (Saure, 2005); the mechanism through which this occurs requires further investigation.

Calcium acts as a secondary messenger downstream of auxin through the acid growth pathway. This pathway has been demonstrated in *Arabidopsis*; auxin eﬄux from cells is facilitated by PIN-FORMED (PIN) membrane proteins. PIN activity and targeted endocytotic transport of PIN are regulated by PINOID (PID) protein kinase and PP2A phosphatase complex mediated phosphorylation (Fozard et al., 2013). Extracellular auxin (possibly though the binding of *ABP1*) activates plasma membrane calcium transport in wheat embryos, creating [Ca^2+^]_cyt_ transients that activate the plasma membrane localized H^+^-ATPase to reduce apoplasmic pH. Lower apoplasmic pH activates pH sensitive cell wall loosening enzymes (Rober-Kleber et al., 2003; Shishova and Lindberg, 2010; Wang and Ruan, 2013). This proton influx into the cell wall compartment also increases competition with calcium for binding sites on de-esterified pectin, resulting in looser cell walls; as such higher levels of calcium can inhibit auxin-activated acid growth (Ferguson, 1984). H^+^-ATPase transport also activates voltage dependent inward rectifying K^+^ channels, this results in an increase in K^+^ content, causing osmotically driven movement of water into the cell, increasing cell turgor pressure. This acid growth pathway is responsible for cell elongation and expansion; it has been observed during growth and hormone-stimulated cell expansion of many tissues. Examples of auxin-calcium interactions in fruit growth are given below.

Auxin is also involved in calcium uptake and distribution in fruit. Application of CME (an auxin transport inhibitor) reduced calcium uptake into developing fruit of some tomato cultivars differing in susceptibility to blossom end rot (Brown and Ho, 1993). This reduced calcium uptake may occur through modification of cellular transport activity or perturbed cell expansion (disrupting xylem development). Calcium is also involved in fruit basipetal auxin transport. CME induced reductions in basipetal IAA eﬄux were only observed in tomato fruit grown under high salinity conditions where calcium uptake was reduced (Brown and Ho, 1993). In kiwifruit, light induction of higher levels of auxin-protecting hydroxycinnamic acids decreased auxin degradation, resulting in increased calcium uptake (Montanaro et al., 2007). These results suggest that tomato susceptibility to blossom end rot may be determined not just by differences in capacity for calcium uptake and distribution, but also by related factors such as auxin transport and metabolism, and rate of cell enlargement (Bangerth, 1976).

### Abscisic Acid

Fruit ripening processes typically involve ABA and ethylene signaling. Non-climacteric fruit show a greater reliance upon ABA for initiation of ripening processes and do not demonstrate the same extent of ethylene responsiveness as climacteric fruit. ABA signaling in *Arabidopsis* acts through a network of calcium binding signal receptors (PYR/PYL/RCAR) and phosphorylation status modifiers including PP2C protein phosphatases ABI1 and ABI2 (Leung et al., 1994; Allen et al., 1999), and an array of CBL (Pandey et al., 2004), CIPK (Kim et al., 2003), and CDPK (Zhu et al., 2007) protein kinases (Fortes et al., 2015). A systems biology approach has been applied to understand the complexity of interactions and crosstalk between these networks (Cramer et al., 2011).

The concentration of ABA in grapes increases dramatically at the beginning of veraison; it is possible that the drop in cell turgor that occurs at this time triggers increases in ABA content (Castellarin et al., 2011). There are varietal differences in the time during veraison (measured as % color change) at which maximal berry ABA is reached; Merlot (10% color change), Cabernet Sauvignon (50% color change), and Semillon (100% color change; Deytieux-Belleau et al., 2008). The partitioning of ABA between mesocarp and skin shifts from 100% in mesocarp prior to veraison, to approximately 40% in skin by maturity (Deytieux-Belleau et al., 2008). In non-climacteric fruit (e.g., strawberries and grapes) several other factors have been identified as potential ripening signal elements; auxin treatment of unripe fruit delays ripening (Boettcher et al., 2011) whilst reactive oxygen species accumulate in grape berries at the onset of ripening (Pilati et al., 2014). The transduction of these ripening triggers through the calcium signaling network suggests that calcium also plays a role in sugar accumulation and fruit softening and evidence for these functions are examined below.

Higher ABA levels at ripening leads to hexose accumulation through up-regulation of hexose transporters and increased apoplasmic invertase activity (Pan et al., 2005; Deluc et al., 2009; Hayes et al., 2010). ABA activates sugar cell wall bound invertases at the initiation of grape ripening, catalyzing sucrose cleavage, decreasing the apoplasmic sucrose concentration, and thereby allowing for continued phloem unloading of sucrose into the berry apoplasm (Pan et al., 2005). Phloem unloading of sugars becomes crucial for driving expansion, as well as efficiently maintaining the accumulation of sugars in the fruit. ABA and sugar responsive elements involved in these pathways have been identified, including an ABA and sugar responsive protein (VvMSR1) that forms part of a complex regulating expression of monosaccharide transporter VvHT1 (Cakir et al., 2003). Microarray expression analysis of cells overexpressing an ABA response element binding transcription factor (VvABF2) demonstrate elevated transcript levels of a vacuolar invertase, a hexose transporter, and cell wall modifying genes linked to fruit softening (i.e., polygalactuonase, pectin methyl esterase and rhamnogalacturonase; Nicolas et al., 2014). It has been demonstrated that ABA activates the calcium dependent protein kinase (ACPK1) in grape mesocarp through a complex mechanism involving influx of apoplasmic calcium to the cytosol (Yu et al., 2006). ACPK1 in turn activates plasma membrane H^+^-ATPase in the berry mesocarp, possibly energizing the cell for solute uptake (Yu et al., 2006). A transient decrease in calcium concentration is observed in the apoplasm of *Vicia faba* leaves following ABA treatment, providing further evidence for apoplasmic calcium as a transducer of ABA signaling (Felle et al., 2000). The timing of ABA accumulation, metabolic responses and the drop in turgor varies between varieties with differing ripening profiles (Deluc et al., 2009; Castellarin et al., 2011).

### Combined Effects of Hormones

Endogenous hormone levels influence fruit softening by altering expression levels of enzymes that modify cell turgor pressure, apoplasm solute accumulation, and cell wall modification. Application of GA has been shown to increase berry firmness and shelf life in the table grape variety Thompson Seedless (Marzouk and Kassem, 2011). Suppression of a key enzyme involved in tomato ABA biosynthesis (9-*cis*-epoxycarotenoid dioxygenase) resulted in the transcriptional down regulation of polygalacturonase, pectin methylesterase, expansion, and many other cell wall modifying enzymes (Sun et al., 2012; Osorio et al., 2013). In Cabernet Sauvignon berries treated with sucrose or sucrose and ABA, a drop in berry firmness (as occurs at the onset of ripening in the field) was only observed in the sucrose and ABA treated berries (Gambetta et al., 2010). The combined effect of ABA-activation of sugar invertases and cell wall modifying enzymes in the apoplasm is to simultaneously reduce cell turgor pressure and loosen cell walls (Pan et al., 2005; Gambetta et al., 2010). Auxin and ABA pathways utilize calcium as both a protein binding secondary messenger and in membrane transport mechanisms that modify turgor and solute accumulation to drive cell expansion and ripening.

## Implications of Calcium Nutrition for Fruit Disease Susceptibility

Understanding the role of calcium in fruit development is important for addressing ripening disorders (e.g., berry shrivel in Shiraz grapes), tissue localized calcium deficiencies (e.g., blossom end rot in tomatoes, bitter pit in apples), and pathogen susceptibilities (e.g., botrytis). Improved understanding of the calcium nutritional requirements of plants may also aid in optimizing fruit quality outcomes as both calcium deficiency and toxicity can affect the productivity of horticultural systems, and the post-harvest characteristics of the crop. Calcium deficiency can occur due to an insufficient mobilization of calcium from internal stores or a reduced supply of calcium through the xylem (often a result of low transpiration rates; White and Broadley, 2003). Calcium toxicity can occur due to high concentrations of available calcium in the soil solution; this can result in reduced growth rates and the ectopic deposition of calcium oxalate crystals (White and Broadley, 2003).

An ABA deficient tomato mutant (*sitiens*; which exhibits botrytis resistance) exhibits a lower degree of epidermal cell wall pectin de-esterification, reduced cuticle thickness, and increased cuticle permeability, when compared to wild type (Asselbergh et al., 2007; Curvers et al., 2010). The consequent reduction in botrytis susceptibility of *sitiens* may be as a result of: (a) plant detection of defective cuticle, prompting constitutive expression of chitinases and β-glucosidases into the cell wall, enabling rapid release of fungal elicitors upon infection, and/or (b) a lower level of de-esterification in *sitiens* cell walls providing a source of more bio-active OGA elicitors upon infection; thereby producing a more rapid and effective response to pathogen attack (Curvers et al., 2010). The level of esterification in OGAs is one of several factors that determine their activity and specificity in triggering plant responses; it has been shown that the level of de-esterification in strawberry OGAs contributes to their capacity to elicit defense responses (Osorio et al., 2008). Although the exact mechanism of botrytis resistance in *sitiens* is unknown, it is clear that the interaction between epidermal cell wall derived pectins and the cuticle, either as defense signaling OGAs or as structural components, is important. In addition to the processes controlling deposition of cutin into the cuticle, processes affecting polysaccharide solubilisation and movement into the cuticle, such as pectin de-esterification and calcium crosslinking, will modify fruit mechanical properties and pathogen susceptibility.

Blossom end rot in tomatoes is often cited as being a result of calcium deficiency. Tomatoes grown in low calcium nutrient solution show an increase in the incidence of blossom end rot (Coolong et al., 2014). Pericarp elasticity increased with calcium levels in the growth solution (Coolong et al., 2014). GA treatment of tomatoes leads to increased occurrence of blossom end rot while treatment with GA biosynthesis inhibitor prohexadione-calcium eliminated blossom end rot (de Freitas et al., 2012). GA treated tomatoes showed increased expression of CAX and Ca-ATPase genes and reduced apoplasmic [Ca^2+^], whereas GA inhibitor treated fruit showed higher pericarp total calcium levels and an increased number of functional fruit xylem vessels (de Freitas et al., 2012). GA-induced gene expression for *CAX* and putative endoplasmic reticulum localized Ca-ATPase results in depletion of the apoplasmic calcium pool, possibly below the critical concentration required for pectin-calcium crosslinks in the cell wall to maintain membrane stability and moderate cell expansion. Similarly, constitutive expression of an *Arabidopsis* CAX gene with its autoinhibitory region removed (sCAX1) in tomatoes led to increased calcium accumulation in the fruit pericarp, but lower calcium levels in apoplasm and cytosol compartments (de Freitas et al., 2011). The sCAX1 line showed leakier plasma membranes, with 100% of fruit demonstrating blossom end rot by 15 days after pollination (de Freitas et al., 2011), highlighting the need for targeted approaches to address localized calcium deficiencies. In addition to the localized decrease in calcium concentration, rapidly expanding tissue (such as the blossom end of tomatoes) may further impede normal fruit calcium distribution due to cellular intrusions causing obstruction or breakage of xylem vessels (Drazeta et al., 2004; de Freitas et al., 2012). Apogee-treated fruit showed increased numbers of functional xylem vessels; the impact of GA on xylem differentiation and development modifies normal pathways for calcium distribution (Saure, 2005). Additionally, GA triggers increased cuticle deposition (Knoche and Peschel, 2007), potentially modifying fruit water relations and calcium uptake. All of these factors provide possible linkages between GA responses and changes in calcium localization leading to blossom end rot.

Other examples of complications arising from sub-optimal calcium nutrition occur in apples and melons. Calcium accumulation in apples is also reduced by progressive breakdown of xylem connectivity as the result of growth related damage, potentially increasing occurrence of bitter pit disorder (Drazeta et al., 2004). In contrast, dye studies in post-veraison grapes indicate that the xylem not only remains relatively intact, but also continues to develop and mature (Chatelet et al., 2008). Application of exogenous calcium has also been proposed as a way to increase apple sugar content and post-harvest shelf life. However, the relationship between calcium and sugar accumulation is complex and many factors appear to affect the effectiveness of this strategy including soil calcium availability, timing of spray/application, apple variety, tree calcium status and *in planta* interactions with other ions (e.g., boron; Lu et al., 2013). Levels of apple tree shading also have a complex effect, with conflicting reports as to whether apple calcium uptake is increased or decreased with shading (Chen et al., 1997; de Freitas et al., 2013). Melons suffer from a water-soaking condition that has been linked to apoplasmic calcium deficiency where it has been hypothesized that depletion of apoplasmic calcium supply can lead to insufficient pectin crosslinks in the middle lamella of the mesocarp, resulting in water-soaked tissue (Madrid et al., 2004; Nishizawa et al., 2004).

## Future Perspectives

Whilst advances in the understanding of water relations in the fruit vasculature are being made, interactions between water and specific extracellular domains are still largely uncharacterised. This review has discussed much of the existing literature that explores the interplay between cell wall composition, calcium binding, and water movement through plants. The observed diversity of ripening patterns demonstrates that, even within a species, inferences from these studies should be made with caution when looking at different species, varieties, or conditions.

**Target areas for further research**

How do calcium-pectin interactions affect water movement through fruit xylem vasculature? Are there critical ‘control points’ in the apoplasm that contribute to fruit water or nutrient deficiencies?

How does the developmental switch from phloem to xylem unloading of solutes affect apoplastic calcium levels, cell wall properties and membrane integrity?

How can our knowledge of calcium delivery, calcium-pectin binding conditions, and calcium signaling pathways during ripening be utilized to address calcium deficiency disorders and improve pathogen resistance?

The relative contributions of xylem and phloem to fruit water influx (or loss) are still a subject of contention. Studies of phloem flows and sap composition are notoriously difficult due to the fine and fragile nature of the compartment. Structural changes, the influence of ionic interactions, and osmotic effects within the xylem also make it a complex and dynamic compartment. It is hoped that a more holistic approach which incorporates not only measures of bulk tissue water balance and molecular mechanisms, but also knowledge of osmotic effects, changes in calcium distribution, pectin gels, and diffusion barriers, will help understand some of the idiosyncrasies of fruit water relations during ripening.

Further studies of calcium distribution in the cell wall and xylem vessels would increase our comprehension of the interactions between calcium nutrition, cell wall processes, and berry water relations. Techniques utilizing fluorescent and luminescent chemical or genetic indicators (e.g., Fluo-4, aequorin, CaR-GECO1 and pHlourin) could be used for quantifying calcium and pH differences (and treatment responses) across different fruit cell types. These techniques have already been applied in other plant tissues (e.g., pollen tubes) to characterize the role of transport and signaling pathway components (e.g., CDPKs; Michard et al., 2008). Application of microscopy techniques for mapping ion concentrations and histochemical localization of cell wall component modifications throughout fruit ripening would also be beneficial. Particularly, combining calcium localization at a sub-cellular level using X-ray microanalysis, or equivalent techniques, with localization of esterified and de-esterified pectins using antibody probes could describe patterns of calcium movement and accumulation in fruit as well as identifying the location of calcium-pectin binding and gel formation (Conn et al., 2011). These results could be correlated with physical properties of fruit (e.g., fruit firmness, elasticity, and skin strength) determined by standard and high throughput methodologies on materials testing devices. This type of approach would bridge the gap between understanding of molecular mechanisms of ion transport and cell wall modification, and observations of fruit physiology impacts on harvest and post-harvest traits.

As the molecular mechanisms of calcium and water transport across cellular membranes are elucidated, and more RNA expression studies in particular fruit and cell types become available (e.g., Fasoli et al., 2012; Sato et al., 2012; Karlova et al., 2014; Palumbo et al., 2014), understanding of the influence of molecular mechanisms on pathways of water and calcium distribution will be improved. Additionally, these studies would help to describe the expression of genes involved in the developmental and stress-induced changes to cell wall composition and modification. This includes elucidation of transcription factor controls, pathogen responses through OGA release from the cell wall and binding by WAKs. The transduction of hormonal signals through calcium dependent kinase networks is also gaining more attention; translation of the functions of these networks in fruit will be an important future development. With this data in hand, a more informed comprehension of the relationships between different components of these pathways will be established.

Although many of the individual roles of calcium in fruit are now being demonstrated, the effect of changes in calcium nutrition on fruit development, susceptibility to pathogens and calcium-related disorders is still lacking. The importance of calcium nutrition in determining susceptibility to major horticultural disorders has been established. However, the amelioration of these disorders and improvement in pathogen resistance through calcium fertilization does not deliver reliable results. Further studies that modify calcium nutrition without affecting other ionic interactions may improve the understanding of optimum plant calcium nutrition and enable better strategies for avoiding fruit physiological disorders and improving fruit physical traits at harvest.

## Author Contributions

BH wrote the majority of the manuscript with input from MG. MG, ST, and RB supervised BH’s Ph.D. from where this review originated. All authors edited and commented on the manuscript.

## Conflict of Interest Statement

The authors declare that the research was conducted in the absence of any commercial or financial relationships that could be construed as a potential conflict of interest.

## Abbreviations

ABA: abscisic acid
Ca^2+^: Calcium ion
CEC: Cation exchange capacity
GA: Gibberellic acid
IAA: Indole acetic acid
K^+^: Potassium ion
OGA: Oligogalacturonide
PME: Pectin methyl-esterase
R_h_: hydraulic resistance
WAK: Wall associated kinase
